# Research on High-Precision and Wide-Range Spacecraft Potential Measurement Method Based on Capacitive Voltage Division

**DOI:** 10.3390/s24237583

**Published:** 2024-11-27

**Authors:** Hong Yin, Haibo Liu, Xiaogang Qin, Qing Liu, Jun Wang, Xuan Wen, Peng Wang, Zixin Yu, Shengsheng Yang

**Affiliations:** Science and Technology on Vacuum Technology and Physics Laboratory, Lanzhou Institute of Physics, Lanzhou 730000, China

**Keywords:** capacitive voltage division, spacecraft potential, space charging, output drift, potential detector

## Abstract

The charging and discharging of satellite surfaces induced by the space plasma environment constitute a primary cause of spacecraft anomalies, particularly in geosynchronous orbits subject to geomagnetic substorms and hot plasma injections from the magnetotail, where satellites are prone to unequal high-potential charging, significantly impacting the safe and reliable operation of spacecraft. Addressing the need for measuring these unequal charge states, a high-precision, wide-range spacecraft potential measurement method based on capacitive voltage division was investigated. This study analyzed the mechanism of potential measurement and the factors contributing to errors during the measurement process, explored optimal design methodologies, and innovatively developed a fundamental charge zeroing method to resolve output drift issues caused by accumulated errors fundamentally. Consequently, a non-contact potential measurement system was developed, featuring a measurement range of up to −15,000 V, a resolution below 15 V, and a nonlinear error of less than 0.1%. This system provides technical support for monitoring the potential state of spacecraft and ensuring their safety and protection.

## 1. Introduction

In the space environment, factors such as plasmas, solar radiation, high-energy particles, and the geomagnetic field collectively interact with orbiting spacecraft, leading to charge accumulation on the surface or within the materials of the spacecraft. This accumulation induces charging and discharging effects that can impair the smooth execution of space missions [1,2,3,4]. According to the U.S. Satellite Anomaly Database, out of 2802 anomaly events recorded in the Geostationary Orbit (GEO) between December 1973 and April 1989, 964 incidents were triggered by electrostatic discharges due to charging, accounting for 34.4% of the total. In 2007, the National Aeronautics and Space Administration (NASA) compiled data from four authoritative institutional databases, revealing that charging and discharging effects were responsible for 54.2% of the 326 satellite failures attributed to the space environment abroad [4,5]. Charging and discharging impacts have become a primary cause of spacecraft malfunctions.

Due to differences in the dielectric properties, illumination conditions, and geometric shapes of satellite surface materials, potential differences can arise between adjacent outer surfaces, between the surface and deeper layers, and between the surface and the spacecraft ground. When these potential differences reach a certain level, discharges can occur in the form of corona, arcing, or breakdown, emitting electromagnetic pulses (EMPs) that can interfere with, damage, or even destroy the spacecraft’s power system, electronic equipment, control system, and its structure or materials. For example, in the Earth’s shadow region, the charging potential on the satellite surface can reach −10 kV. During the transition out of the shadow region, parts of the spacecraft exposed to tenuous plasmas and solar illumination will charge to a few to several tens of volts positively, while unilluminated parts can still reach tens of thousands of volts. This significant unequal charging poses a considerable safety hazard to satellites [6]. Additionally, suspended components, such as cables, when accumulated with charged particles, can form unequal charged potential differences with the spacecraft body, posing a certain risk of discharge. Henry B. Garrett mentioned that breakdown will occur if the electric field magnitude exceeds the breakdown strength of the surrounding space or if the electric field at the interface between the surface dielectric and exposed grounded conductor is higher than 10^5^ V/cm (NASA TP-2361) [7]. Therefore, measuring the charging level of spacecraft is extremely necessary and crucial, providing essential data for the safety assessment of satellite surface charging, unequal charging, and active and passive potential control protection.

The primary methods for measuring the surface potential of spacecraft currently in orbit include the vibrating capacitance method (tuning fork method), resistive voltage divider method, capacitive voltage divider method, probe measurement method, and low-energy ion spectroscopy method, among others (Table 1). Among these, the capacitive voltage divider method, which employs indirect capacitance measurement technology, is widely used in space potential measurement payloads due to its simple structure, small size, low power consumption, and adaptability to both high-orbit and low-orbit environments. It has been implemented in various spacecraft, such as the INTELSAT series [8,9,10], multiple communication satellites of the NSS series [11], the P78-2 satellite [12], and the GSAT-2 satellite [13], as well as China’s Fengyun-4 satellite and the low-Earth-orbit Chinese Space Station [14].

However, during operation, environmental noise, such as thermal effects, can influence the capacitance value and voltage division ratio, thereby limiting the measurement range or accuracy of this method. For instance, the INTELSAT series can measure potentials within the range of −1500 V to 0 V [9,10], while the Fengyun-4 satellite was designed to measure up to 3 kV but with a resolution of 20 V [14]. Presently, achieving a wide dynamic range and high-resolution potential measurement, particularly for the high charging potentials in Earth’s shadow region, remains a significant technical challenge.

This study focuses on advancing high-precision, wide-range potential measurement using the capacitive voltage divider method. It investigates the factors affecting measurement accuracy and proposes a rapid and thorough charge neutralization method to address output drift issues during probe operation. Additionally, this study optimizes the backend circuitry and conducts ground-based simulation experiments. The objective of our potential detector is to provide a high-precision spacecraft potential measurement solution with a range exceeding tens of kilovolts, thereby supporting subsequent spacecraft potential evaluation and active potential control through reliable data.

## 2. Analysis and Design of Spatial Potential Detectors

### 2.1. Principles of Potential Measurement

The physical process of charging for spacecraft in orbit is the result of surface current balance, influenced by the combined effects of space plasma, charged particles, and photons. The potential $V_{p}$ on various surfaces of the spacecraft is determined by its current balance equation as follows:

(1) $$I_{T}\left(V_{p}\right)=I_{e}\left(V_{p}\right)+I_{i}\left(V_{p}\right)+I_{se}\left(V_{p}\right)+I_{si}\left(V_{p}\right)+I_{b}\left(V_{p}\right)+I_{ph}\left(V_{p}\right)$$

In the equation, $I_{T}\left(V_{p}\right)$ denotes the spacecraft current (which is 0 in equilibrium), while $I_{e}\left(V_{p}\right)$ and $I_{i}\left(V_{p}\right)$ represent the incident electron current and ion current on the spacecraft surface, respectively. $I_{se}\left(V_{p}\right)$ is the secondary electron current induced by $I_{e}\left(V_{p}\right)$, $I_{si}\left(V_{p}\right)$ is the secondary electron current induced by $I_{i}\left(V_{p}\right)$, $I_{b}\left(V_{p}\right)$ is the backscattered electron current induced by $I_{e}\left(V_{p}\right)$, and $I_{ph}\left(V_{p}\right)$ denotes the photoelectric current. According to the Mott–Smith and Langmuir Orbit Motion Limited (OML) theory, the charging current densities $J_{e}\left(V_{p}\right)$ and $J_{i}\left(V_{p}\right)$ on the spacecraft surface within the space plasma are given as follows [21]:

(2) $$J_{e}=\frac{qN_{e}}{2}\sqrt{\frac{2kT_{e}}{\pi m_{e}}}e^{\frac{qV_{p}}{kT_{e}}}$$

(3) $$J_{i}=\frac{qN_{i}}{2}\sqrt{\frac{2kT_{i}}{\pi m_{i}}}\left(1-\frac{qV_{p}}{kT_{i}}\right)$$

In the equation, *q* represents the unit charge; $N_{e}$ and $N_{i}$ denote the number densities of charging electrons and ions, respectively, at a surface potential of 0 V; $T_{e}$ and $T_{i}$ represent the electron and ion temperatures; and $m_{e}$ and $m_{i}$ denote the masses of electrons and ions. By utilizing local plasma parameters along with Equations (1)–(3), the surface potential of a spacecraft in different orbital plasma environments can be estimated.

**Figure 1:**
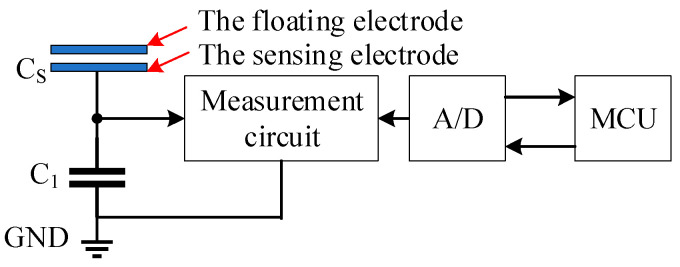
Block diagram of the potential detector system.

For the in-orbit measurement of spacecraft potentials, the capacitive voltage divider-based method enables the measurement of the differential charging potential between various surfaces. As shown in Figure 1, the potential detector consists of two main components: the sensor and the measurement circuit. The sensor is formed by two series-connected capacitors: one structural capacitor *Cs*, comprising a floating electrode and a sensing electrode, and one standard electronic capacitor *C*_1_. During measurement, the floating electrode of *Cs* is either connected to the spacecraft’s target surface or left floating, while the sensing electrode of *Cs* is connected to the measurement circuit, which includes an A/D data acquisition and processing circuit and a host computer for measurement processing. The voltage on the sensing electrode is proportional to the voltage on the floating electrode. When measuring the relative potential *Cs* between the spacecraft surface and the satellite structure, the floating electrode of *Cs* is connected to the spacecraft surface, and one terminal of *C*_1_ is connected to the satellite structure ground. In this configuration, the relationship is given by the following:

(4) $$V_{s}=V_{p}-V_{ref}$$

When measuring the differential charging potential $V_{ds}$ between different surfaces of the spacecraft, multiple sensors can be deployed. These sensors measure the relative potentials of various parts or surfaces of the spacecraft. By calculating the differences between these relative potentials, the differential charging potential $V_{ds}$ between different surfaces can be determined. This approach allows for comprehensive monitoring of uneven charging conditions across the spacecraft’s surfaces.

(5) $$V_{ds}=V_{s1}-V_{s2}$$

In this equation, $V_{s1}$ and $V_{s2}$ represent the relative potentials of different surfaces of the spacecraft. Using two sensors installed on the sunlit and shadowed surfaces of the spacecraft, the difference between their measurements can be calculated to obtain the relative potential difference between these surfaces. This enables an assessment of the differential charging conditions on the spacecraft’s surface. Such measurements provide critical data for evaluating the risks of spacecraft charging and discharging, ensuring the safety of satellite docking operations, and supporting astronaut extravehicular activities.

### 2.2. Capacitive Voltage Divider Network Model

**Figure 2:**
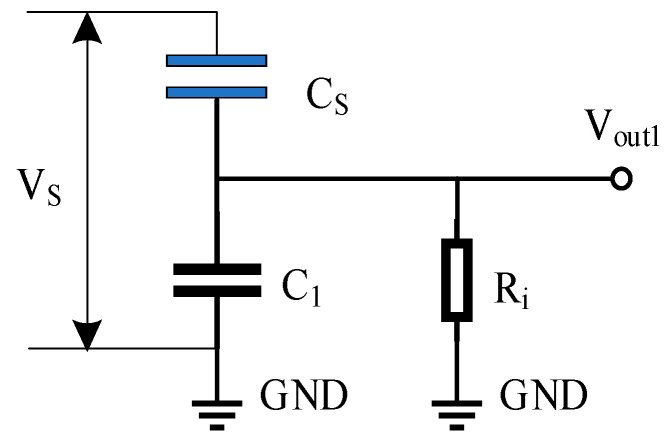
Basic model of a capacitive voltage divider network.

The equivalent capacitive voltage divider network model is illustrated in Figure 2, where $V_{s}$ represents the surface voltage to be measured and $V_{out1}$ represents the output voltage measured by the divider network.

According to Kirchhoff’s Current Law (KCL), the current flowing through *C_S_* is equal to the sum of the currents through *C*_1_ and the resistor *R_i_* as follows:

(6) $$C_{s}\frac{d\left[V_{s}(t)-V_{out1}(t)\right]}{dt}=\frac{V_{out1}(t)}{R_{i}}+C_{1}\frac{dV_{out1}(t)}{dt}$$

By integrating the above equation, we obtain the following:

(7) $$V_{s}(t)=\frac{C_{1}+C_{s}}{C_{s}}V_{out1}(t)+\frac{1}{R_{i}C_{s}}\int_{0}^{t}V_{out1}(t)dt$$

(8) $$V_{out1}(t)=\frac{C_{s}}{C_{1}+C_{s}}exp\left[-\frac{1}{R_{i}(C_{1}+C_{s})}\right]V_{s}(t)$$

It is evident that there exists a time constant tending towards stability between $V_{out1}$ and $V_{s}$, with this time constant being $\tau =R_{i}(C_{1}+C_{s})$. Over short periods, the exponential term has minimal influence on $V_{out1}$, and $V_{out1}$ exhibits a linear positive proportional relationship with $V_{s}$ as follows:

(9) $$V_{out1}(t)=\frac{C_{s}}{C_{1}+C_{s}}V_{s}(t)$$

### 2.3. Exploration of Factors Affecting the Accuracy of Potential Measurement

**Figure 3:**
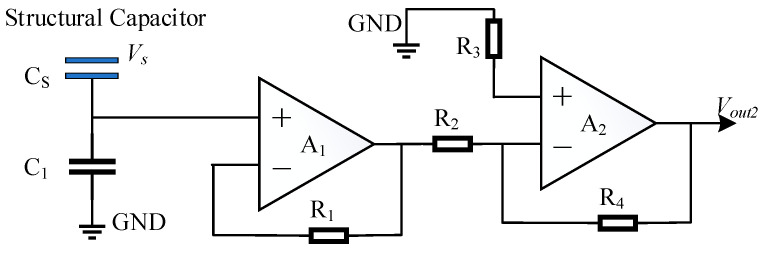
Equivalent circuit diagram of the potential measurement probe.

The spacecraft surface potential detector design features an equivalent circuit depicted in Figure 3. In this circuit, the structural capacitance *Cs* is formed by coupling two metal plates (a floating electrode and a sensing electrode), which are separated by a high-resistance polyimide spacer. A_1_ represents a voltage follower circuit characterized by its high input impedance and low output impedance. This circuit not only achieves impedance matching but also serves as a signal buffer and isolator. A_2_ is an amplification circuit responsible for increasing the amplitude or power of the input signal, thereby enhancing signal quality. The relationship between the surface voltage $V_{S}$ to be measured, and the output voltage $V_{out2}$ of the detector is given as follows:

(10) $$V_{out2}=V_{S}\cdot \frac{C1+C2}{C1}\cdot K$$

In the equation, K represents the amplification factor of the amplification circuit.

**Figure 4:**
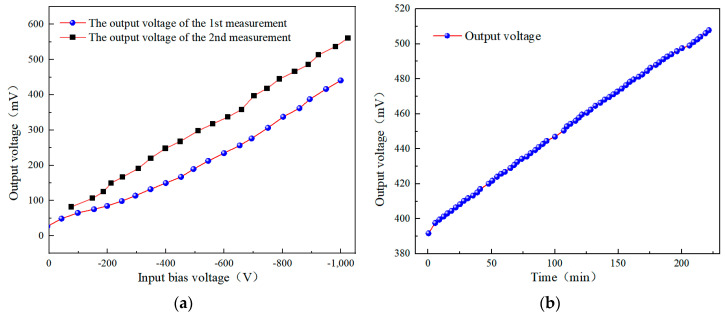
Probe output drift results: (**a**) comparison of probe output results across multiple measurements; (**b**) drift of probe output results over time with −1 kV bias input.

Using the detector designed above for potential measurement experiments, Figure 4 presents the output results of the probe from two measurements. It can be observed that the two measurement results are inconsistent (Figure 4a), indicating poor repeatability in potential measurement. Furthermore, when a bias voltage of −1 kV is applied to the floating electrode, the potential output signal $V_{out2}$ exhibits significant output drift. As time accumulates, the output drift becomes more pronounced, leading to a notable increase in output error (Figure 4b).

The main factors affecting the output drift, which impacts the accuracy of potential measurement, include the following.

(1) Influence of Input Bias Current

The non-inverting input of the operational amplifier A_1_ is directly connected to the voltage divider capacitor. When measuring negative potential V_S_, the input bias current of the circuit will flow back to the voltage divider capacitor, causing voltage accumulation at the capacitor terminals and resulting in output drift.

(2) Influence of Leakage Current

Leakage current is a significant factor affecting electrostatic potential measurement, and its formation is complex. It includes leakage currents from the sensor to the structural ground, leakage currents in the voltage divider capacitor, leakage currents from the input lines to the ground, leakage currents from the operational amplifier input terminal’s static voltage relative to the power supply and ground, and leakage currents between relay contacts and lines. Leakage current continuously leaks induced charges to the ground, leading to output drift.

(3) Influence of Ambient Temperature

Temperature affects material size expansion strain and component performance, thereby causing changes in structural capacitance and component accuracy. According to thermal expansion characteristics, polyimide materials have a thermal expansion coefficient of 2 × 10^−5^, and a 50 °C change in operating temperature can cause a thermal expansion strain of 1 × 10^−3^. Ambient temperature also influences the performance of the detector’s backend circuit components. As ambient temperature increases, the probe’s leakage current also increases. In specific designs, data compensation can be applied through experimental calibration to address the impact of temperature on output.

(4) Influence of Dielectric Materials

The floating electrode, the sensing electrode, the probe housing, and the structural ground are isolated from each other through high-resistance polyimide insulating pads. However, polyimide materials are not completely insulating, and there is still a certain leakage current between the sensor probe and the spacecraft structure. Additionally, dielectric materials, like polyimide, can exhibit polarization under high-voltage electric fields [22], resulting in degraded insulation, which significantly affects the stability and accuracy of potential measurement. During the design process, polyimide dielectrics exist directly between the floating electrode and the sensing electrode, and this should be avoided.

(5) Influence of Discharge Between Structural Capacitors

When there is a high potential difference between the sensing electrode of the structural capacitor, charges on the high-potential plate may discharge through the polyimide insulating pad to metal fasteners, causing creeping discharge and damaging the insulation performance of the polyimide pad. There is also a risk of edge discharge and arc discharge between the two plates, which can directly cause the failure of the capacitive voltage divider measurement function. During the design process, the creepage path should be increased to prevent creeping discharge, and the insulation installation spacing between the two plates should be reasonably designed. The two plates should be kept clean with smooth edges, without burrs or sharp points, to avoid discharge between the metal plates.

### 2.4. Design of Potential Detector

(1) Electronics Design

**Figure 5:**
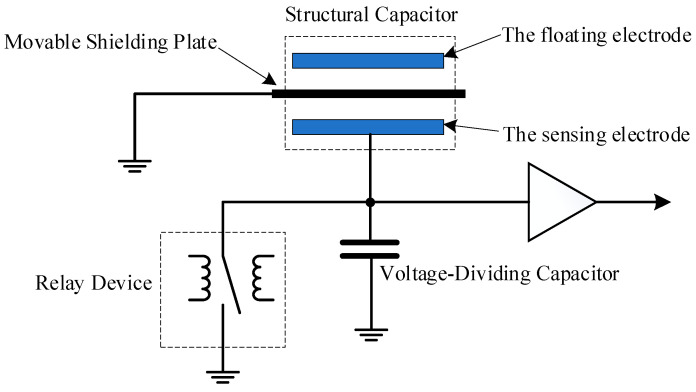
Schematic of the electrostatic charge zeroing circuit.

Addressing the issue of output drift mentioned above, a series of optimization methods was implemented during the design process of the potential measurement probe, including adding a bias circuit, increasing input impedance, enlarging the exponential term constant, correcting temperature data, and optimizing insulation installation. However, the problem of leakage current caused by the structure or circuit components cannot be completely avoided, which results in cumulative output errors as measurement time progresses. Regularly eliminating the accumulated charges on the sensing electrode through charge zeroing is an effective approach to thoroughly resolve the output drift issue. Nevertheless, simply short circuiting the sensing electrode to the structural ground results in an induced electric field formed by the potential difference between the floating electrode and the sensing electrode, exerting an electric field force on the charges on the sensing electrode. This prevents the accumulated charges on the sensing electrode from being fully released to the structural ground. To address this problem, the team specifically introduced a movable metal shielding plate between the floating electrode and the sensing electrode. Before potential measurement or after a certain period of measurement, the shielding plate is positioned between the floating electrode and the sensing electrode (Figure 5) and grounded, disrupting the electric field between the floating electrode and the sensing electrode. As both the shielding plate and the sensing electrode share the same ground, the charges on the sensing electrode can be fully released.

**Figure 6:**
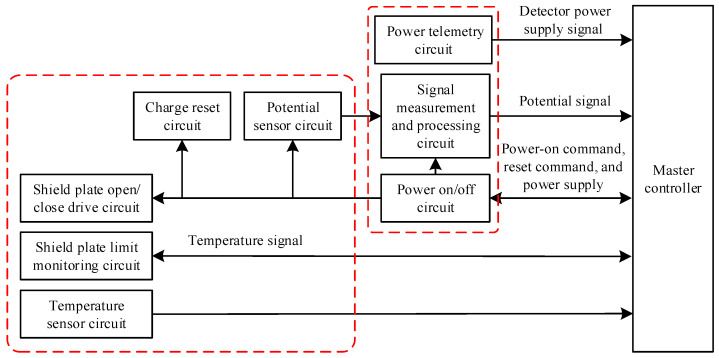
Structural design of the potential detector circuit.

The designed potential probe circuit system includes a potential measurement circuit, a shielding plate opening/closing drive circuit, a zeroing circuit, and a temperature acquisition circuit, as shown in Figure 6.

The potential measurement circuit consists of a voltage follower circuit, an inverting amplifier circuit, and an output clamping circuit, providing functions such as high-impedance input, voltage amplification, and low-pass filtering. A low-bias current operational amplifier is employed as the input stage, minimizing charge leakage on the voltage divider capacitor and achieving high-impedance input. Simultaneously, signal paths, including the voltage divider capacitor and the operational amplifier input terminal, are enclosed and isolated by equipotential shielding to prevent leakage currents introduced by the circuit medium. A predetermined voltage division ratio is achieved by setting appropriate resistor values, and low-pass filtering is applied to reduce circuit noise, ensuring measurement accuracy.

The shielding plate opening/closing drive circuit utilizes an electromagnet to drive the shielding plate, pushing and pulling it between the floating electrode and the sensing electrode.

The zeroing circuit operates by pushing the shielding plate between the two plates and subsequently zeroing the charges on the sensing electrode via a relay. A controller powers it, and the power interface incorporates a restrictive backflow protection measure to avoid high pulse currents in the circuit.

The temperature acquisition circuit is used for the calibration of the potential sensor. By collecting the temperature inside the sensor, a certain temperature compensation correction is applied to the potential sensor, enabling the measured potential to eliminate the influence of temperature.

(2) Design of Composition Modules

**Figure 7:**
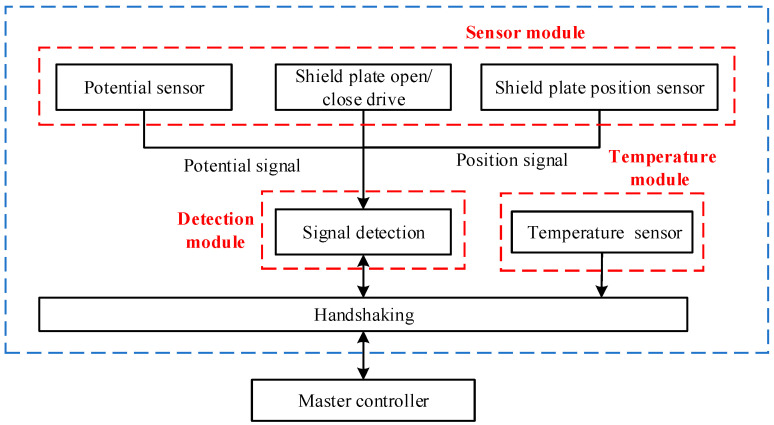
Functional modules of the potential measurement probe.

As shown in Figure 7, the designed potential measurement probe primarily consists of a mechanical module, a potential measurement module, a shielding plate position measurement module, and a temperature measurement module.

The functional modules of the potential measurement probe comprise a shielding plate opening/closing drive mechanism, a position sensor, and a potential sensor. Before measuring unequal electric potentials, charge zeroing is performed first. The drive mechanism actuates the shielding plate to initiate zeroing of both the sensor probe and the circuit. The position sensor enables the perception of the drive device’s operational status while the potential sensor processes and transmits potential signals between the sensor probe and the controller.

During the potential measurement process, the potential measurement probe operates in three modes: shutdown mode, charge zeroing mode, and potential measurement mode. In shutdown mode, all power inputs are disconnected. Before potential measurement, the charge zeroing mode is engaged. In this mode, the control host sends commands to the detection circuit in a timed sequence, prompting the movable mechanism to actuate the shielding plate, positioning it in the middle of the structural capacitance. Subsequently, the relay operates, short circuiting the sensing electrode to the structural ground for approximately 3 s and then disconnecting the ground. The shielding plate returns to its original position, completing the entire charge zeroing operation of the probe. Upon the completion of charge zeroing, the probe receives commands and transitions into potential measurement mode.

In practical application, considering the electromagnetic interference (EMI) between the potential detector and other onboard equipment, as well as the impact of the external space radiation environment on the detector, the detector can be arranged internally within the spacecraft. The floating electrode can be connected to the surface of the component under test via shielded cables, and metallic housing can be used to effectively shield electromagnetic interference from other equipment. Additionally, effective grounding, electromagnetic compatibility (EMC), and electromagnetic interference (EMI) testing can be implemented to improve the design, enhance the detector’s electromagnetic compatibility, and increase its resistance to interference, ensuring reliable operation of the detector in complex space environments.

## 3. Experimental Testing of the Detector

### 3.1. Ground Simulation Test System and Protocol

**Figure 8:**
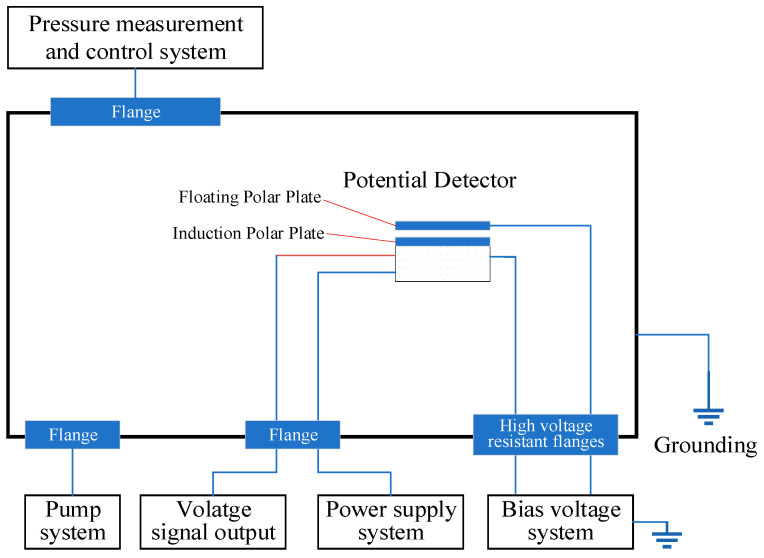
Test system for the potential measurement probe.

As shown in Figure 8, to ensure the reliability and stability of the potential measurement probe, ground simulation tests and calibrations were conducted to verify its potential detection function and zero-setting performance. The specific tests were carried out in a vacuum environment, with the vacuum environment simulation equipment capable of reaching an ultimate vacuum level of 10^−4^ Pa. As illustrated, the potential measurement probe was suspended inside the vacuum chamber, and the cables were connected between the interior and exterior of the chamber through a flange. The input voltage lines of the probe’s electrode plates were connected via high-voltage-resistant flanges.

**Figure 9:**
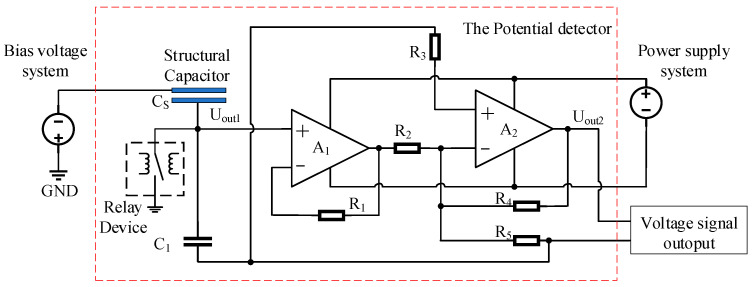
Equivalent circuit diagram for potential measurement.

The equivalent circuit diagram used in the experiment is shown in Figure 9. The negative terminal of the bias voltage system was connected to the floating electrode plate of the probe, while the ground terminal was connected to the probe’s housing and the internal circuit ground, simulating the unequal charged potentials between the spacecraft structure and the suspended components. The internal circuit of the potential measurement probe was powered and controlled by the power supply system, and the collected voltage signals were output and measured by a digital multimeter.

### 3.2. Analysis of Experimental Results

**Figure 10:**
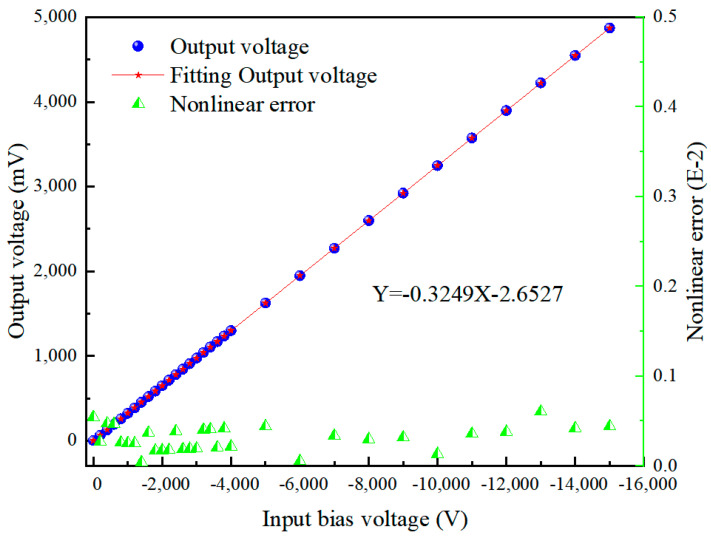
Experimental calibration results of the potential measurement probe.

**Figure 11:**
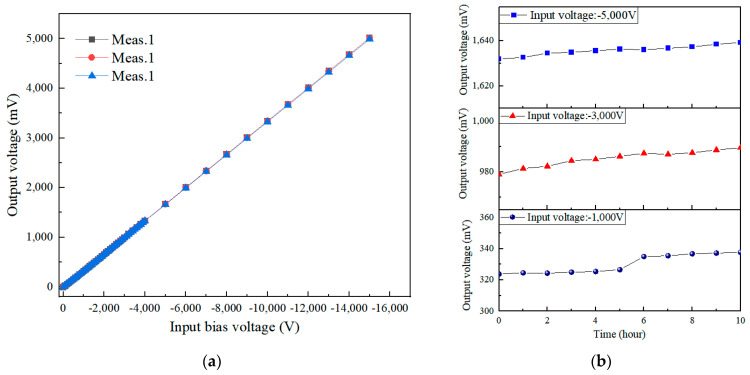
Results of probe output drift: (**a**) comparison of probe output results across multiple measurements; (**b**) drift of probe output results over time with −1 kV, −3 kV, −5 kV bias voltage inputs.

Figure 10 and Figure 11 present the output voltage characteristics of the improved detector after performing the zero-setting operation. Figure 10 reveals a good linear relationship between the output voltage and the input bias voltage. By conducting a linear regression analysis on the input–output data, we obtained a fitting curve, which can serve as the calibration curve for the detector. Specific data are provided in Table 2. Further analysis of the linear error in Figure 10 shows that the maximum nonlinear error of the probe is controlled within 0.1%. It can be seen that the improved detector exhibits good output linearity. (Here, the nonlinear error is defined as the maximum deviation between the corrected output–input curve and its fitted linear curve, typically expressed as the relative error $\Upsilon_{V}=\left(∆V/V_{Fs}\right)\times 100\%$, where ∆V represents the maximum nonlinear deviation of the output and $V_{Fs}$ is the full-scale output).

Figure 11 shows a comparison of the output results from multiple measurements of the detector and the drift of the detector’s output over time. As can be seen in Figure 11a, the improved detector exhibits good repeatability in measurement output after zero setting, and its output changes very little over time, indicating excellent stability. There is no sudden increase in a short period, as shown in Figure 11b. This demonstrates that the zero-setting operation can effectively release the accumulated electrostatic charges in the probe and circuit, thereby improving the measurement accuracy of the detector.

## 4. Conclusions

Addressing the need for on-orbit potential measurement of satellites, this study focused on resolving the issue of output drift in potential measurements using the capacitive voltage divider method. A non-contact, high-precision potential measurement detector was developed, and a simulated space potential measurement experiment was conducted to verify the measurement stability and reliability of the detector.

The experimental results of the potential detector demonstrated that after performing the zero-setting operation, the detector exhibited a good linear relationship between the output voltage and the input bias voltage. It can initially achieve high-precision voltage measurement within the range of −15,000 V to 0 V, with a measurement accuracy of less than 15 V and a nonlinear error of the output of less than 0.1%. The detector demonstrated high output accuracy and long-term operational stability. Various measures were incorporated into the structural and electronic design of the detector to eliminate leakage currents, such as the use of high-resistance insulating materials in the structural design, the addition of a charge zeroing mechanism, and the adoption of a bias circuit, which increased input impedance, exponential term constants, and temperature data correction in the circuit design.

The data indicate that the technological innovations in this study significantly improved the accuracy and reliability of spacecraft potential measurements, providing strong technical support for the safe execution of space missions.

## Figures and Tables

**Table 1:** Comparison of current spacecraft potential measurement methods and applications.

Measurement Method	Principle	Advantages	Disadvantages	Typical Applications
Vibrating Capacitance Method (Tuning Fork Method) [15]	Measures potential via tuning fork probe vibrations and capacitance changes.	High sensitivity, suitable for low-potential measurements.	Vibration sensitive, structurally complex, limited adaptability.	
Resistive Voltage Divider Method	Measures potential via resistive voltage division.	Simple circuit, easy to implement.	Sensitive to noise, with high power consumption.	
Capacitive Voltage Divider Method	Spacecraft potential is indirectly measured via a spatial capacitance voltage divider network.	Non-contact, low power, adaptable to diverse space environments.	Prone to environmental noise, limited accuracy, requires calibration.	INTELSAT series; NSS series; GSAT-2; Chinese Space Station; Fengyun-4.
Probe Measurement Method [16]	Directly measures surface potential via probe sensing or contact with surface charges.	High sensitivity, directly measures spacecraft surface potential.	Susceptible to contamination or damage, limited probe lifespan	Comet 67P [17].
Low-Energy Ion Spectroscopy Method [18]	Infers spacecraft potential from ion spectra–plasma interactions.	Provides detailed plasma parameters and potential variations.	The equipment is complex, relatively large in size, power intensive, and slow to respond.	SCATHA; DSCS satellite [19,20].

**Table 2:** Experimental calibration data for the potential measurement probe.

Input Bias Voltage (V)	Output Voltage (mV)	Fitting Output Voltage (mV)	Input Bias Voltage (V)	Output Voltage (mV)	Fitting Output Voltage (mV)
0	0	−2.65	−3200	1039	1037.03
−200	61	62.33	−3400	1104	1102.01
−400	125	127.31	−3600	1168	1166.99
−600	190	192.29	−3800	1234	1231.97
−800	256	257.27	−4000	1298	1296.95
−1000	321	322.25	−5000	1624	1621.85
−1200	386	387.23	−6000	1947	1946.75
−1400	452	452.21	−7000	2270	2271.65
−1600	519	517.19	−8000	2598	2596.55
−1800	583	582.17	−9000	2923	2921.45
−2000	648	647.15	−10,000	3247	3246.35
−2200	713	712.13	−11,000	3573	3571.25
−2400	779	777.11	−12,000	3898	3896.15
−2600	843	842.09	−13,000	4224	4221.05
−2800	908	907.07	−14,000	4548	4545.95
−3000	973	972.05	−15,000	4873	4870.85

## Data Availability

Data are contained within the article.
